# Topics of Public Interest

**Published:** 1910-02

**Authors:** 


### TOPICS OF PUBLIC INTEREST.

### Reasons for Rushing the Municipal Hospital

**A New Case of Contagious Disease Every Twenty Minutes—Delay Costs Over$1,000 a Day in Money, Besides Suffering, Sickness and Death—Best Street Site Suitable—Ferry Street Site Unsuitable.**

After stating that a majority of the board are in favor of a Convention Hall on Broadway; that all believe the Best street site inaccessible for a high school and that another site for the school can be found in a location more central for the students, the committee recommends immediate action on the project for a municipal hospital because of other considerations, and continues:

REPORT OF COUNCILMEN JANUARY 19, 1910.

These considerations are the alarming condition in regard to contagious diseases, and the imperative need for a hospital for the proper treatment of them. Diphtheria, scarlet fever and measles are the principal contagious diseases to be treated at the proposed hospital. Cases of these three diseases in 1908 were 4,828, an average of over 13 per day, or a new case for every two hours day and night throughout the year. This was more than half of the total number of births during the entire year, and four-fifths the number of deaths; that is, for every two babies that were born there was a new case of one of these contagious diseases, and for every five people who died there were four cases of contagious diseases.

The figures for 1909 show the frightful effect of delay and inaction in facing this tremendous problem. The total cases of these three diseases increased to 7,484, more than 20 a day, nearly one new case every hour throughout the year, 1,300 more than all the deaths in the city from all causes during the same period, and only a little less than the total number of births. During December the figures ran up to 2,553, more than 83 a day, and more than one for every 20 minutes.

The Health Department has furnished an estimate of loss to the people of Buffalo through contagious diseases for two years past. Estimating the cost to the family of scarlet fever and diphtheria at $50 a case, measles at $20, and a funeral $150, the figures are $215,000 for 1908, and $315,000 for 1909. For December, 1909, on the same basis they would be over $60,000, not counting funerals. Even cutting these figures in two, the present direct money loss to the people from these three diseases must be over $1,000 a day.

The matter of a hospital for contagious diseases was intrusted last year to a commission composed of the Mayor, Commissioner of Public Works and Health Commissioner, constituting the Board of Health, with the presidents of the Common Council,

Board of Councilmen and Board of Aldermen, and the Chairman of the Committee on Sanitary Measures. This commission reported last April in favor of the Best street site for a hospital. After being requested by the Aldermen to consider the subject further, the Commission, on October 21, after a public hearing of the opposition, adhered to its preference for the Best street site. In a later report of the Committee on Sanitary Measures, at page 2791, the only reason given for not purchasing the Best street site was that the city owns sufficient land on Ferry street adjoining the smallpox hospital, and the committee considered that site preferable.

Dr. Wende, our efficient Health Commissioner, in a strong official statement, at pages 2825 and 2826, shows conclusively that the Ferry street site is absolutely unfit for consideration from the medical and sanitary point of view, and strongly urges the Best street site.

It is true that some residents near the Best street site object to a hospital there. This sort of objection in greater or less degree is urged to the erection of every hospital, school house and fire building. It has been increased in the present instance by loose talk about a pest house. This hospital will not take smallpox cases.

The cases in the contagious hospital will be measles, diphtheria, scarlet fever, whooping cough, chicken pox and spinal meningitis, with all of which the people of Buffalo are thoroughly familiar through sad experience. It will be no more a detriment to property than the contagious wards of the Sisters, the General and the Children's Hospital. The city has had a scarlet fever hospital for temporary use in a condemned school building on Broadway for over a year, without the slightest objection or complaint by any neighbor.

Recognising these facts, the trustees of the German Roman Catholic Orphan Asylum, which owns the property, are willing to sell it and to retain their present institution on the other side of Dodge street. These trustees are well known philanthropic citizens, taking good care of their institution, and they see no valid objection to the hospital being built just across the street from their asylum. The doctors of the city are practically a unit in favor of this site.

We present herewith separate resolutions carrying out these views.

(Signed)

Alfred H. Burt,<br>

L. Bradley Dorr,<br>

Jacob J. Siegrist,<br>

Charles L. Willert,<br>

Henry Adsit Bull,<br>

John T. Mahoney,<br>

H. H. Bingham,<br>

Wm. Burnet Wright, Jr.,

Statement by Dr. Wende, Cited in Above Report.

Buffalo, N. Y., November 18, 1909.

*To the Committee on Sanitary Measures, Buffalo, N. Y.:*

Gentlemen: I have previously, whenever occasion demanded, expressed my views on the matter of a hospital for contagious diseases, and I again desire to urge immediate action towards the disposition of a question the neceessity of which has long been universally recognised and whose absence is becoming a source of grave public danger.

Buffalo has outgrown all the resources which were adequate for a primitive condition, and our citizens have a right to demand proper municipal equipments in the care and treatment of contagious diseases which shall at least be equal—and ought to be superior—to the best modern standard.

From September 1, 1908, to October 31, 1909, we have had in this city, 3,890 cases of scarlet fever, 754 cases of measles and 994 cases of diphtheria, while the records thus far in November show an undue increase both in number and virulence, with a mortality above the average. The city's lack of facilities in meeting the demands to care for these cases is well known to your honorable commission.

I, therefore, urge upon you to dispose of the site question so that the succeeding steps may be taken in their proper order.

In connection herewith, I desire to say, that of all the sites offered for this purpose, I consider the Best street site superior in every respect. Its geographical location, altitude, soil and other general conditions commend themselves.

In my opinion there is no danger from the hospital to the surrounding inhabitants, and far less danger than ordinarily exists in densely populated districts.

It is also my judgment that the greatest error will be committed, and the city will always regret the step taken, if the hospital for acute contagious diseases be erected on the Ferry street property, now owned by the city. The very fact that the hospital for smallpox is located on these grounds will deter a majority of people from permitting their children or relatives to go there, and no amount of argument will convince them that there is no danger of infection from this source. It was never an ideal spot for a hospital of any kind, but no one connected with the present administration of the city's affairs is responsible for that. In addition to the open sewer, in the shape of a creek, flowing past, a great proportion of the lot and for a considerable distance around it is "made ground," filled in with various kinds of city dumpings and refuse, and honey-combed in a way to form a natural abode for innumerable rats. These are some of the main objections which, in my opinion, render the Ferry street site unsuitable for this purpose.

The abolition of a smallpox hospital is likewise out of the question. The necessity for it is always urgent. Without it the city would not only be frequently put to great inconvenience and expense, but would also have the distinction of standing alone among cities in the absence of such facilities. Even at the present time we have three patients there under treatment. By means of this hospital we are enabled to immediately remove the patient, thus preventing the spread and rendering indefinite quarantine of others unnecessary.

Owing to systematic vaccination, Buffalo was fortunate in this respect, for the total number of cases in the United States during the past three months was 3 974.

Signed ERNEST WENDE, M. D.,
Health Commissioner.

---

### Millions Spent in Tuberculosis Crusade

**Statistics of Ten States Given—New York in the Lead—Survey of Year's Work affords Interesting Figures.**

BASED on reports gathered from all parts of the United States, the National Association for the Study and Prevention of Tuberculosis issues a bulletin today in which it is stated that $8,180,621.50 was expended during the year just closed by the various interests fighting consumption in the United States. The bulletin, which is preliminary to a longer report, shows that in the year 1909 over 10 000,000 pieces of literature were distributed, and that 117,312 patients were treated and assisted by the sanatoria, dispensaries and anti-tuberculosis associations.

By far the largest amount of money spent during the past year was for the treatment of tuberculous patients in sanatoria and hospitals, $5,292,289.77 being expended in this way. The anti-tuberculosis associations spent $975,889.56, the tuberculosis dispensaries and clinics, $640,474.64, and the various municipalities, for special tuberculosis work, spent $1,111,967.53. The anti-tuberculosis associations distributed the most literature, spreading far and wide 8,400,000 copies of circulars, pamphlets, and other printed matter for the purpose of educating the public about consumption. The health departments of the different cities also distributed more than 1,056,000 copies, which, with the work done by state departments of health, brings the number of pieces distributed during the year well over 10 000,000. The largest number of patients treated during the year was by the dispensaries, where 61,586 patients were given free treatment and advice. The sanatoria and hospitals treated 38,758 patients, while anti-tuberculosis associations assisted 16,968.

New York State leads in the anti-tuberculosis work done during the past year, having spent more money, distributed more literature and treated more patients than any other state. Pennsylvania comes next and Massachusetts is third. The next seven states are Illinois, Maryland, New Jersey, California, Colorado, Connecticut and Ohio. The following table shows the work done in these ten states:

| State | Expenditures | Literature, pieces distributed | Patients treated |
|---|---|---|---|
| New York ............. | $1,669,179.76 | 4,997,600 | 41,779 |
| Pennsylvania .......... | 1,515,664.02 | 251,300 | 24,410 |
| Massachusetts ......... | 1,059,123.53 | 217,605 | 10,645 |
| Illinois ................. | 202,820.53 | 254,500 | 4,826 |
| Maryland ............. | 195,691.07 | 29,500 | 5,829 |
| Ohio ................... | 245,502.17 | 127,000 | 3,197 |
| New Jersey ............ | 211,660.62 | 287,500 | 2,159 |
| Colorado ............... | 566,205.17 | 37,000 | 3,229 |
| California ............. | 254,707.14 | 107,075 | 1,900 |
| Connecticut ........... | 220,190.98 | 13,500 | 1,141 |

Although the survey of the past year's work shows that much has been done, the reports from all parts of the country indicate that next year the amount of money to be expended, and the actual number of patients that will be treated will be more than double that of the past year. For instance, special appropriations have been made in the various municipalities for next year's anti-tuberculosis work, aggregating $3,976,500. In addition to these appropriations over $4,000,000 has been set aside by the different state legislatures for the campaign against tuberculosis next year. Besides these sums, a large number of the present existing institutions and associations are planning enlargements of their work, and new organisations are being formed daily.

---

At the dinner of the Metropolitan Life Insurance Company at the Hotel Astor a few nights ago, announcement was made of the donation of ninety acres of land on a high elevation in the State of New York, with $100,000 in cash, to the company for the purpose of erecting a sanatorium for the care and cure of tuberculous clerks and agents of the company. The donor is in no way connected with the company, and was moved to make the offer by the difficulties the company has had in securing legal authority to put up the sanatorium.

The dinner was given by the company to commemorate the thirtieth anniversary of the beginning of industrial insurance, and the completion of its large home office building and tower and in honor of Mr. Pierre Le Brun, the architect, who is retiring from the active practice of his profession.

A magnificent clock of silver, modeled after the Metropolitan tower, including the chimes, made by Tiffany & Co., was given to Mr. Le Brun in commemoration of the completion of his work. The donor of the sanatorium was present but refused to have his name mentioned. He is a leading capitalist of the city.

